# Generation of *GLA*-Knockout Human Embryonic Stem Cell Lines to Model Autophagic Dysfunction and Exosome Secretion in Fabry Disease-Associated Hypertrophic Cardiomyopathy

**DOI:** 10.3390/cells8040327

**Published:** 2019-04-08

**Authors:** Hui-Yung Song, Chian-Shiu Chien, Aliaksandr A. Yarmishyn, Shih-Jie Chou, Yi-Ping Yang, Mong-Lien Wang, Chien-Ying Wang, Hsin-Bang Leu, Wen-Chung Yu, Yuh-Lih Chang, Shih-Hwa Chiou

**Affiliations:** 1 Institute of Pharmacology, National Yang-Ming University, Taipei 11221, Taiwan; shy770307@gmail.com (H.-Y.S.); cschien6688@gmail.com (C.-S.C.); yarmishyn@gmail.com (A.A.Y.); ohyeahchou@gmail.com (S.-J.C.); monglien@gmail.com (M.-L.W.); 2 Department of Medical Research, Taipei Veterans General Hospital, Taipei 11217, Taiwan; molly0103@gmail.com (Y.-P.Y.); hbleu@vghtpe.gov.tw (H.-B.L.); 3 School of Medicine, National Yang-Ming University, Taipei 11221, Taiwan; wangcy@vghtpe.gov.tw (C.-Y.W.); wcyu@vghtpe.gov.tw (W.-C.Y.); 4 School of Pharmaceutical Sciences, National Yang-Ming University, Taipei 11221, Taiwan; 5 Department of Emergent Medicine, Taipei Veterans General Hospital, Taipei 11217, Taiwan; 6 Division of Cardiology & Department of Medicine, Taipei Veterans General Hospital, Taipei 11217, Taiwan; 7 Department of Pharmacy, Taipei Veterans General Hospital, Taipei 11217, Taiwan; 8 Genomics Research Center, Academia Sinica, Taipei 11574, Taiwan

**Keywords:** Fabry disease, human embryonic stem cells, CRISPR/Cas9 genomic editing, Mass spectrometry proteomic analysis, hypertrophic cardiomyopathy, disease model

## Abstract

Fabry disease (FD) is a rare inherited disorder characterized by a wide range of systemic symptoms; it is particularly associated with cardiovascular and renal problems. Enzyme replacement therapy and pharmacological chaperone migalastat are the only approved and effective treatment strategies for FD patients. It is well documented that alpha-galactosidase A (GLA) enzyme activity deficiency causes globotriaosylceramide (Gb3) accumulation, which plays a crucial role in the etiology of FD. However, the detailed mechanisms remain unclear, and the lack of a reliable and powerful disease model is an obstacle. In this study, we created such a model by using CRISPR/Cas9-mediated editing of *GLA* gene to knockout its expression in human embryonic stem cells (hESCs). The cardiomyocytes differentiated from these hESCs (GLA-null CMs) were characterized by the accumulation of Gb3 and significant increases of cell surface area, the landmarks of FD-associated cardiomyopathy. Furthermore, we used mass spectrometry to compare the proteomes of GLA-null CMs and parental wild type CMs and found that the Rab GTPases involved in exocytotic vesicle release were significantly downregulated. This caused impairment of autophagic flux and protein turnover, resulting in an increase of reactive oxygen species and apoptosis. To summarize, we established a FD model which can be used as a promising tool to study human hypertrophic cardiomyopathy in a physiologically and pathologically relevant manner and to develop new therapies by targeting Rab GTPases signaling-related exosomal vesicles transportation.

## 1. Introduction

Approximately 3–5% of inherited hypertrophic cardiomyopathies result from lysosome storage disorders [1]. One of these cardiomyopathies is Fabry disease (FD), which is caused by alpha-galactosidase A (GLA) deficiency leading to accumulation of globotriaosylceramide (Gb3) in several tissues. FD is particularly manifested in renal and cardiovascular dysfunctions [2]. A number of in vivo and in vitro studies revealed that the loss of GLA results in left ventricular hypertrophy and develops into heart failure, myocardial infarction and life-threatening arrhythmias due to Gb3 deposition. The levels of Gb3 and its deacylated derivative, globotriaosylsphingosine (lyso-Gb3) in plasma and tissues are used as diagnostic biomarkers of FD, and are applied for the screening/monitoring of FD patients by liquid chromatography-tandem mass spectrometry (LC-MS/MS) [3,4,5,6]. However, the limited sample volume and variable levels of lyso-Gb3 in different matrices makes the quantitation very challenging [7]. Furthermore, the detailed mechanism of how Gb3 accumulation results in hypertrophic cardiomyopathy still needs to be elucidated. In order to develop new therapeutic strategies for FD-associated vasculopathy, it is essential to understand the underlying pathogenesis mechanisms, as well as to discover the potential prognostic biomarkers.

Previously, we generated CRISPR/Cas9-edited GLA-null human HEK293 cells; however, this cell line may not be an appropriate model with which to study pathological events occurring in cardiomyocytes [8]. The pluripotent stem cells, including embryonic stem cells (ESCs) and induced pluripotent cells (iPSCs), offer a great potential for modelling human diseases as they can be differentiated into the tissue affected by the pathology. Although several FD-specific iPSC lines have been obtained from patients carrying different *GLA* gene mutations, which claimed to be useful for FD cardiomyopathy research [9,10], one major limitation of such approach is the influence of variable genetic background, which can be significant even for monogenic, dominant and highly penetrant disease in FD [11,12]. Currently, CRISPR/Cas9 emerges as a powerful genome editing technique, providing the opportunity to efficiently delete genes to establish isogenic cells [13,14,15]. Therefore, our strategy in this study was to generate *GLA* knockout in human pluripotent stem cells by CRISPR/Cas9-mediated gene editing, and compare them with the parental cells of the same genetic background to study the mechanisms of FD-associated cardiomyopathy. 

Recently, it became increasingly clear that lysosomal storage disorders have an impact on autophagic dysfunction [16]. Dysregulated ceramide metabolism can trigger cytotoxic signaling cascades, including apoptosis and necroptosis, missorting and accumulation of these sphingolipids in the membrane subdomains may destabilize lipid bilayer and cause their permeabilization [17]. Exosomes are small vesicles secreted upon fusion of multivesicular endolysosomal compartments with the plasma membrane and are derived from the intraluminal vesicles (ILVs) of those organelles. Exosomes may participate in the control of cellular homeostasis by promoting the release of intracellular harmful components, including proteins, lipids or nucleic acids. Emerging evidence from the studies of normal development, as well as multiple disease studies, is beginning to reveal a coordinated exosome–autophagy response that functions to maintain homeostasis through lysosomal degradation and release of cellular cargo [18,19,20]. However, little effort has been made to investigate the impact of autophagic dysfunction in FD on biogenesis and secretion of exosomes. 

In the current study, we applied CRISPR/Cas9-mediated genomic editing to deplete *GLA* expression in ESC-derived cardiomyocytes to recapitulate FD cardiac hypertrophy in vitro and performed proteomic analysis by LC-MS/MS. We identified that Rab GTPase signaling-related vesicle secretion is the factor that may initiate or exacerbate the development of FD-associated cardiomyopathy. Such information will be extremely important for potential application in the prevention of and in interventions for the adverse effects of the cardiac hypertrophy in FD patients.

## 2. Materials and Methods

### 2.1. CRISPR/Cas9 Plasmid Construction and Transfection

The CRISPR/Cas9 with T2A-eGFP co-expression vector pSpCas9(BB)–2A-GFP (PX458) was a gift from Feng Zhang (Addgene plasmid). The exon 1 of *GLA* was selected for guiding RNA design and the sequence (5′-AGGAACCCAGAACTACATCT-3′) was cloned into PX458 (abbreviated as GLA-Cas9-GFP) as previously described [8]. The *GLA*-specific targeting plasmid was transfected into hESC line (WA09) by electroporation using Nucleofector System (Lonza, Basel, Switzerland) following the manufacturer’s protocol. Briefly, hESC cells were cultured to 80–90% confluence, then harvested and washed with PBS without Ca^2+^ and Mg^2+^. Approximately 4 × 10^5^ cells were resuspended in the pre-mixture solution with 2 μg GLA-Cas9-GFP plasmid, and the optimized protocol (program B016) was used for electroporation. The cells were plated on Matrigel-coated 6-well plate in mTeSR1 medium containing 10 μM Y27632. 48 h later, the proportion of cells expressing EGFP was enriched by flow cytometry using FACSCalibur (BD Biosciences, San Jose, CA, USA). Three days later, cells were detached with TrypLE (Invitrogen, Thermo Fisher Scientific, Waltham, MA, USA), separated into single cells and seeded with a density of 1 cell/well of a 96-well dish.

### 2.2. Analysis of CRISPR/Cas9-Induced Mutations in GLA Gene

To identify the presence of indels in *GLA* gene, the genomic DNA was extracted and used for PCR amplification of the target site with the primer pair 5′-CACACACCAACCTCTAACGATACC-3′ (forward) and 5′-CCAGGAAAGGTCACACAGAGAAAG-3′ (reverse). PCR products were TA-cloned into pGEM-T Easy vector (Promega, Madison, WI, USA). Subsequently, DNA from the clones #19 and #27 was sequenced using T7 forward and Sp6 Reverse primer. Vector NTI software was used to align the results of sequencing and determine the indel spectra in *GLA* target site.

### 2.3. hESC Culture and Differentiation to Cardiomyocytes

The hESCs were cultured on tissue culture dishes coated with Geltrex (Life Technologies, Thermo Fisher Scientific) in mTeSR1 culture medium (STEMCELL Technologies, Vancouver, BC, Canada) with daily media changes. The cells were passaged every 3–4 days using Accutase (STEMCELL Technologies). The undifferentiated phenotype of the hESCs was checked daily using a light microscope. In order to differentiate hESCs to cardiomyocytes, they were dissociated by Versene (Life Technologies, Thermo Fisher Scientific), resuspended in mTeSR1 + 5 μM Y27632 and seeded onto Geltrex-coated plates at a density of 3 × 10^5^ cells/cm^2^ and grown for the next four days with daily medium change. Following that, the cells were treated with 6 µM CHIR99021 (Selleckchem, Houston, TX, USA) in insulin-free RPMI/B27 medium (Life Technologies) for 24 h. The medium was replaced with basal medium for another 2 days. At day 3, the culture medium was subsequently replaced with 5 µM IWP-2 (Tocris Bioscience, Minneapolis, MN, USA) in insulin-free RPMI/B27 for 48 h. On day 7, the culture medium was changed to RPMI/B27 containing insulin (Life Technologies, Thermo Fisher Scientific), and the culture medium was refreshed every 3 days thereafter. 

### 2.4. Alkaline Phosphatase Staining

Cells were washed in PBS twice, fixed with 80% alcohol for at least 2 h at 4 °C, followed by soaking in ddH_2_O for 2–3 min, and 100 mM Tris-HCl (pH 8.2–8.5) for 5 min. Alkaline phosphatase substrate working solution (Vector Laboratories, Burlingame, CA, USA) was added for 1 h and stained colonies were visualized under light microscope.

### 2.5. Reverse Transcription-Polymerase Chain Reaction (RT-PCR)

Total RNA was isolated with TRIzol reagent (Invitrogen, Thermo Fisher Scientific) and quantified by spectrophotometry at 260 nm. 3 μg of total RNA was reverse transcribed using SuperScript III Reverse Transcriptase (Invitrogen, Thermo Fisher Scientific) at 55 °C for 1 h into complementary DNA, which was then used as the template for subsequent PCR reactions. The PCR reactions were run with the following cycling conditions: 94 °C for 5 min, followed by 25 or 30 cycles at 94 °C (denaturation) for 30 s, 58–62 °C for 30 s (annealing), 72 °C for 45 s (synthesis). The primer sequences are shown in Supplementary Tables S1 and S2. Amplified RT-PCR products were analyzed on 2% agarose gels and visualized using ethidium bromide staining and SPOT camera system (Diagnostic Instruments, Sterling Height, MI, USA).

### 2.6. Immunofluorescence Staining

First, the cells were rinsed in PBS and fixed with 1% (*v*/*v*) paraformaldehyde for 10 min followed by treatment with 70% ethanol (*v*/*v*) for 10 min at room temperature. The cells were permeabilized with 0.1% NP-40 (Sigma-Aldrich, St. Louis, MO, USA) for 20 min, then washed twice with PBS. To block cells, blocking solution (0.3% BSA and 5% serum in PBS) was applied for 30 min. Cells were incubated with primary antibodies in the blocking solution overnight at 4 °C, washed three times in PBS, then stained with secondary antibodies at 1:200 in PBS for 1 h at room temperature. Cells were washed three times in PBS, and nuclei were stained with Hoechst 33,342 (Life Technologies, Thermo Fisher Scientific) at 1:5000 in PBS for 5 min at room temperature. Prior to imaging, cells mounted with SlowFade Gold Antifade Mountant (Millipore, Sigma, Burlington, MA, USA). Images were obtained using fluorescent microscopy and a digital camera. Antibody for characterization of pluripotency is listed in Supplementary Table S3.

### 2.7. Measurement of Cardiomyocyte Size

The size of iPSC-derived cardiomyocytes was evaluated by measuring the cell area. Twenty days after cardiac induction, the spontaneously beating embryoid bodies were dissociated into single cells using Accutase™ (STEMCELL Technologies). These cells were then plated onto gelatin-coated dishes for further experiments and analysis. Subsequently, the cellular images of cTnT-positive cells were recorded at days 30, 40 and 60 post-induction of differentiation using FV10i confocal microscope (Olympus, Tokyo, Japan). The pixel area of cTnT-positive cells was measured and analyzed using ImageJ software package (NIH). About total two hundred cells were analyzed in five independent experiments.

### 2.8. Western Blotting

Cells were lysed in RIPA lysis buffer (0.5M Tris-HCl, pH 7.4, 1.5M NaCl, 2.5% deoxycholic acid, 10% NP-40, 10 mM EDTA, protease inhibitor), and the protein lysates were subjected to SDS-PAGE followed by electroblotting onto a PVDF membrane. Membranes were probed with the following primary antibodies: α-galactosidase A, TSG101 (GeneTex, Irvine, CA, USA), CD63 (Santa Cruz Biotechnology, Dallas, TX, USA), Calnexin (Abcam, Cambridge, MA, USA) GDRID2, VPS36, VTI1A (Proteintech Group, Wuhan, Hubei, P.R.C), LC3 (Novus Biologicals, Centennial, CO, USA), Rab11 and GAPDH (Cell Signaling Technology, Denver, MA, USA). The bands were visualized by chemiluminescence detection reagents.

### 2.9. Lipid Extraction

The cells were grown to confluency on 150 mm cell culture dishes and harvested by scraping into 700 μL PBS. The cell suspension was transferred into 16 mm × 100 mm glass tubes, and 1 mL chloroform and 2.4 mL methanol were added. After water bath sonication, protein was precipitated by centrifuging at 2400× *g* for 30 min. The supernatant was transferred to a new glass tube and 4.5 mL chloroform and 1.2 mL 0.9% NaCl were added. The sample was centrifuged at 900× *g* for 5 min. The upper aqueous phase was discarded, and the lower organic phase was washed twice with 2 mL methanol and 0.8 mL 0.9% NaCl. The lower phase was extracted using a 1 mL glass syringe (Hamilton, Reno, NV, USA) and transferred to a new glass tube. Lipids were dried under a stream of nitrogen.

### 2.10. Quantification of Gb3 by Thin Layer Chromatography (TLC)

First, 100 nmole of total phospholipid was applied to a silica high performance TLC plate (Sigma-Aldrich, St. Louis, MO, USA). The plate was first developed in a solvent system consisting of chloroform/methanol (98:2), and air-dried. The plate was then developed in a solvent system consisting of chloroform/methanol/acetic acid/water (61/31/5/3) and air-dried. Plates were submerged in 8% cupric sulfate pentahydrate in water/methanol/H_3_PO_4_ (60:32:8), and charred for 10 min at 150 °C, or were sprayed with 1% orcinol in 11% H_2_SO_4_ and charred at 130 °C for 5 min. Plates were scanned and densinometry measured using ImageJ software. Lipids were quantified by running Gb3 standards on a plate (Matreya LLC, State College, PA, USA).

### 2.11. Liquid Chromatography-Tandem Mass Spectrometry (LC-MS/MS) Analysis 

LC-MS/MS analysis was performed using Orbitrap Mass Analyzer (Thermo Fisher Scientific), according to the manufacturer’s protocol. Briefly, each sample of digested peptides was reconstituted in 20 μL of 0.1% formic acid. Peptides were first separated by the nanoflow HPLC on Agilent 1100 (Agilent Technologies, Santa Clara, CA, USA) using C18 column (Agilent Technologies) with a flow rate of 0.4 μL/min, and were ionized after passing through the nanospray tip (New Objective, Woburn, MA, USA). LC gradient for the LC-MS/MS system ramped from 2–40% ACN in 120 min, and the system was set up for automated data-dependent acquisition, with a mode of 200–2000 m/z full scan for the maximum three most intense peaks from each Orbitrap MS scan. Peptides with +2 or +3 charge were further subjected to CID. Spectra were obtained in raw data files with Xcalibur (version 2.0 SR2). Protein identification was accomplished by TurboSEQUEST (Thermo Fisher Scientific) using the UniProt database. A protein was confirmed once three peptides with Xcorr >2.5 were matched in sequencing.

### 2.12. Transmission Electron Microscopy

The morphology of differentiated cardiomyocytes was characterized using JEM-2000 EX II transmission electron microscope (JEOL, Tokyo, Japan). The cardiomyocytes were covered with 400 mesh carbon-coated copper TEM grid. After 15 min, the grid was tapped with filter paper to remove the excess water followed by staining with 1% phosphotungstic acid (Sigma-Aldrich) for 20 min. The samples were allowed to air-dry for 24 h and then observed under TEM.

### 2.13. Array-Based Comparative Genomic Hybridization (CGH-Array

Genomic DNA was isolated and intermittently sonicated using a Digital Sonifier 450 sonicator probe (Branson Ultrasonics, Danbury, CT, USA). DNA samples were amplified using the GenomePlex WGA kit (Thermo Fisher Scientific). Genomic DNA ULS Labeling Kit (Agilent) was used to label the amplified DNA with either Cy3 or Cy5. As recommended by Agilent, 2.0–2.5 μg of amplified DNA was used as the input starting material for each labeling reaction. Scanning and image analysis were conducted according to Agilent Oligonucleotide Array-based CGH for Genomic DNA analysis Protocol (version 4.0). Microarrays were scanned using an Agilent G2565BA DNA Microarray Scanner (Agilent). Agilent Feature Extraction software (v9.1.3) was used to extract data from raw microarray image files. Agilent CGH Analytics software (v3.4) was used to visualize, detect and analyze the aberration patterns from CGH microarray profiles.

### 2.14. Exosome Isolation and Characterization 

Exosomes were isolated from cell culture supernatants using Total Exosome Isolation Reagent (Thermo Fisher Scientific) following the manufacturer’s protocol. Culture media samples were centrifuged at 2000× *g* for 30 min to remove cells and debris. The supernatant was transferred to sterile tubes and an exosome precipitation solution was added at a 2:1 ratio. Samples were mixed and left overnight at 4 °C. Samples were then centrifuged at 10,000× *g* for 60 min and supernatant carefully removed. The precipitated exosome pellets were re-suspended with PBS and either used immediately or stored at −80 °C until required. Immunoaffinity capture assay was used to characterize the purity of exosomes using CD63 antibody-conjugated dynabeads. Exosomes were incubated with antibody-conjugated dynabeads overnight and washed by PBS containing 0.1% BSA two times. Further, CD63 PE-conjugated antibody was used to stain the exosome-bound dynabeads. All samples were examined by flow cytometry using FACSCanto System (BD Biosciences) and FACSDIVA software was used to analyze the population of exosomes. 

### 2.15. Quantification of Isolated Exosomes

Acetylcholinesterase activity assay was performed using EXOCET Exosome Quantitation Kit (System Biosciences, Palo Alto, CA, USA). Isolated exosomes were re-suspended with PBS and lysed with lysis buffer. Each sample was incubated at 37 °C for 5 min and mixed with reaction buffer in 96-well plates. Mixed samples were incubated for 20 min at room temperature and quantified by spectrophotometry at OD 405 using standard samples containing known numbers of exosomes. The final number of exosomes was converted to micrograms, per the manufacturer’s guideline. To characterize exosomes, isolated exosome preparations were re-suspended with PBS and analyzed using Zetasizer Nano dynamic light scattering system (Malvern Instruments, Malvern, UK).

### 2.16. Mitochondrial Superoxide Stress Quantification

Mitochondrial superoxide production was quantified using MitoSOX Red mitochondrial superoxide indicator (Thermo Fisher Scientific) according to the manufacturer’s protocol. Briefly, the cells were incubated with 5 µM MitoSOX Red in the culture medium in the dark for 60 min at 37 °C. Stained cells were counterstained as desired and mounted and analyzed by fluorescent microscopy.

### 2.17. Annexin V Staining

Cells were washed once with PBS and analyzed using FITC Annexin V Apoptosis Detection Kit (BD Biosciences) according to the manufacturer’s protocol.

### 2.18. GLA Enzyme Activity Assay

Cells were washed twice with 1X PBS and were lysed in 60 μL lysis buffer (27 mM sodium citrate, 46 mM sodium phosphate dibasic, 0.5% Triton X-100). 10 μL of cell lysate was added to 50 μL assay buffer containing 6 mM 4-methylumbelliferyl-α-d-galactopyranoside (Sigma-Aldrich) and 117 mM N-acetyl-D-galactosamine (Sigma-Aldrich) and incubated at 37 °C for 1 hr. The 4-methylumbelliferone (Sigma-Aldrich) dissolved in methanol was used as standard ranging from 0.15 μM to 5000 μM. Thereafter, 70 μL glycine-carbonate solution (pH 10.8) was then added to stop the reaction and fluorescence was detected by microplate reader (em/ex = 365/448 nm). The enzyme activity was normalized by protein concentration of cell lysate.

### 2.19. Statistical Analysis

The quantifiable data are presented as the means ± standard deviation (SD) and compared with Student’s *t*-test by GraphPad Prism 6 (GraphPad Prism Software). * *p* < 0.05, ** *p* < 0.01, *** *p* < 0.005 and **** *p* < 0.001.

## 3. Results

### 3.1. CRISPR/Cas9-Mediated Knockout of Expression of GLA in hESCs

**Figure 1:**
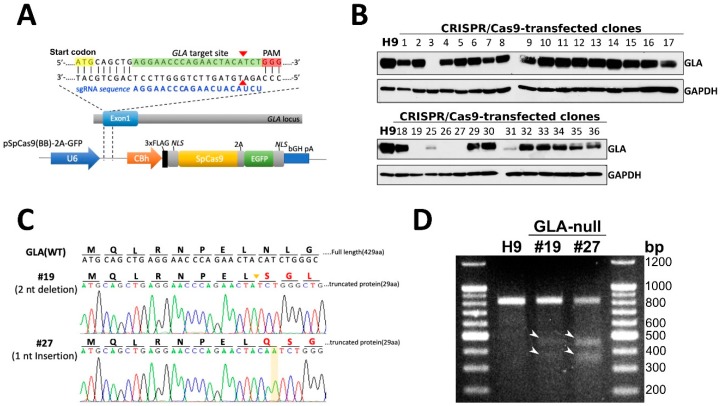
CRISPR/Cas9-mediated knockout of expression of *GLA* in hESCs. (**A**) Schematic depiction of sgRNA-guided Cas9 target site within exon 1 of *GLA* gene. The sgRNA sequence (5′-AGGAACCCAGAACUACAUCU-3′) is labeled in blue font and PAM recognition sequence highlighted in red. The gRNA targeting site in the *GLA* exon 1 region is highlighted in green and the double-strand breaking site is indicated by the red arrowheads. The start codon is highlighted in yellow. (**B**) Western blot screening of 30 CRISPR/Cas9-corrected clones for expression of GLA protein. H9 cells were used as wild type parental control. GAPDH used as a loading control. (**C**) Sanger sequencing analysis confirming two nucleotides deletion and one nucleotide insertion in CRSIPR/Cas9-edited hESC clones #19 and #27, respectively. (**D**) T7E1 digestion assay of the mutants at the target site in *GLA* gene. The mismatch T7E1 cleavage products are marked with white arrowheads.

In order to investigate the functions of *GLA* and its therapeutic potential for FD, we performed CRISPR/Cas9-mediated gene editing to knock out expression of this gene in human embryonic stem cells (hESCs). The procedure was similar to that described in our previous work, where we established the GLA-null HEK293 cell line [8]. Briefly, the *GLA*-specific single-guide RNA (sgRNA) was designed by using Optimized CRISPR Design tool (http://crispr.mit.edu/) to introduce the frameshift mutations in exon 1 of *GLA* gene as shown in Figure 1A. The parental male hESC line H9 (WA09) was transfected with Cas9-encoding plasmid with EGFP reporter, along with the sgRNA. Following the transfection, the proportion of the successfully transfected EGFP-expressing cells was enriched by FACS, and these cells were seeded to 96-well dishes to obtain pure colonies. Out of 36 wells where the single cells were seeded, the clones #20 to #24 and #28 did not grow up. The remaining 30 colonies were screened for GLA protein expression by western blotting (Figure 1B). The GLA protein expression was completely ablated in four clones (#3, #19, #26, and #27) and partially suppressed in two (#25 and #31) (Figure 1B). By performing preliminary differentiation of these clones into cardiomyocytes (CMs), we found that clone #26-derived CMs displayed slight GLA expression, which could be due to contamination with parental non-edited cells, therefore, this clone was excluded from further analysis (Supplementary Figure S1A). *GLA* mRNA levels in these CM clones were not significantly decreased as compared to the control, only in clone #27-derived CMs mRNA levels were downregulated by approximately 50% (Supplementary Figure S1B). On the other hand, GLA enzyme activity was completely absent in clones #3, #19, and #27, which was consistent with the complete absence of GLA protein in these cells (Supplementary Figure S1C). Subsequently, the mutations introduced by CRISPR/Cas9 were identified by Sanger sequencing in the remaining *GLA*-knockout hESC clones #3, #19, and #27. Clone #3 displayed mixed genetic background at the target site, due to putative non-homogeneous population, therefore, it was also excluded from the analysis. (Supplementary Figure S1D). Two of GLA-null hESC clones were selected for the following study because they were hemizygous for 2 bp deletion (clone #19) and 1 bp insertion (clone #27) (Figure 1C). T7 Endonuclease I (T7E1) was used to confirm the presence CRISPR/Cas9-introduced mutations in the *GLA* gene, whereby the cleaved mismatch products were detected in clones #19 and #27 (Figure 1D). On the other hand, T7E1 assay revealed that there were no CRISPR/Cas9-introduced mutations in the potential off-target genes predicted by Optimized CRISPR Design tool (Suppl. 2). Therefore, by conducting the CRISPR/Cas9-mediated genome editing, we successfully established two GLA-null hESC clones.

### 3.2. Characterization of GLA-Null hESC Clones

**Figure 2:**
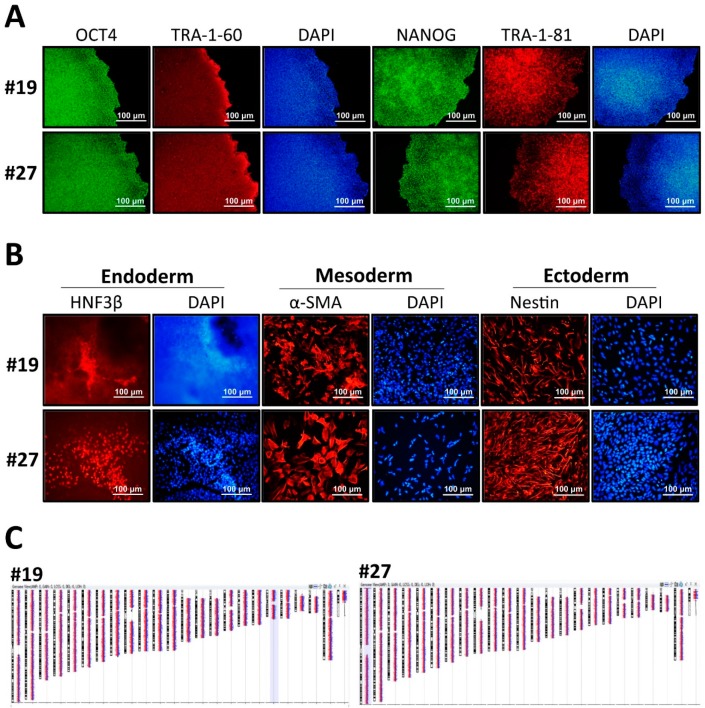
Characterization of CRISPR/Cas9-edited GLA-null hESC clones. (**A**) Immunofluorescence staining demonstrating the protein expression of pluripotency markers OCT4, TRA-1-60, NANOG and TRA-1-81 in GLA-null hESC clones #19 and #27. Nuclei stained with DAPI. (**B**) Embryoid body formation assay showing differentiation of GLA-null hESC clones #19 and #27 into three germ layers: endoderm immunoreactive for HNF3β, mesoderm immunoreactive for α-SMA, and ectoderm immunoreactive for nestin. Nuclei stained with DAPI. (**C**) Representative karyograms GLA-null hESC clones #19 and #27. Visualization was performed using Agilent CytoGenomics software.

To confirm the pluripotency status of the established GLA-null hESC clones #19 and #27, several stemness markers were detected by RT-PCR, thus confirming that CRISPR/Cas9-mediated *GLA* knockout did not significantly influence the pluripotency gene expression in hESCs (Supplementary Figure S3A). Both GLA-null hESC clones displayed typical pluripotent morphology features, including small and tightly packed cells, high nucleus to cytoplasm ratio, and positive alkaline phosphatase activity (Supplementary Figure S3B). The expression of pluripotency markers OCT4, NANOG, TRA-1-60 and TRA-1-81 was verified by immunofluorescence staining (Figure 2A). Both null-GLA hESC clones were able to spontaneously differentiate in vitro into embryoid bodies expressing markers of three germ layers: HNF3β of endoderm, α-SMA of mesoderm, and nestin of ectoderm (Figure 2B). Both clones exhibited normal karyotypes (Figure 2C). To summarize, both established GLA-null hESC clones exhibited normal stem cell properties, and therefore, were suitable for differentiation into FD-specific cardiomyocytes to investigate the mechanisms of involvement of GLA deficiency in the pathology of FD cardiomyopathy.

### 3.3. Recapitulation of FD-Specific Cardiac Abnormalities in GLA-Null Cardiomyocytes

**Figure 3:**
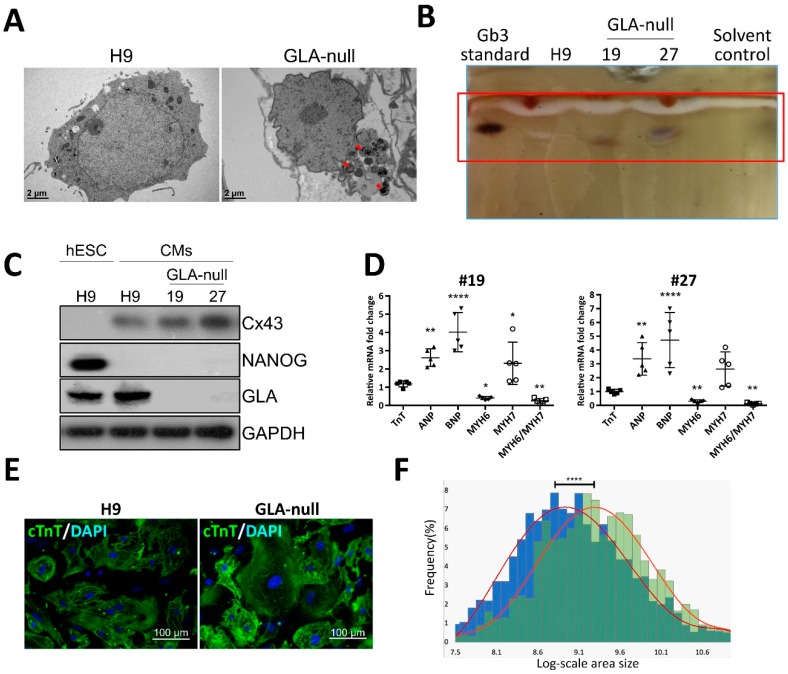
Recapitulation of FD-specific cardiac abnormalities in GLA-null cardiomyocytes. (**A**) TEM images showing the ultrastructure of parental type (H9) and GLA-null CMs. The red arrowheads indicate the multilayered lysosomal structure. (**B**) Gb3 content in parental (H9) and GLA-null CMs (clones #19 and #27) analyzed by TLC. (**C**) Western blot showing lack of expression of GLA in clones #19 and #27 of GLA-null CMs as compared to the parental wild type CMs and hESCs (H9). Connexin 43 (Cx43) and NANOG served as cardiomyocyte and pluripotency markers, respectively. GAPDH used as a loading control. (**D**) qRT-PCR analysis of expression several fetal heart markers in CM clones #19 and #27. The results are expressed as fold change relative to H9 CMs. (**E**) Immunostaining of cTnT showing significantly enlarged size of GLA-null CMs compared to H9 CMs. (**F**) Quantification of area size of GLA-null CMs (green columns) and H9 CMs (blue columns). At least 200 cells were analyzed individually and statistical difference is *p* < 0.001.

Given the fact that CM hypertrophy is one of the symptoms of FD-associated cardiomyopathy, we examined whether GLA-null CMs could recapitulate such features. hESCs were differentiated into CMs according to the conventional CM differentiation protocol [21]. Both GLA-null CMs and CMs derived from the parental H9 hESCs (H9 CMs) exhibited spontaneous contractions as early as twelve days following initiation of cardiac differentiation. Noticeably, no significant differences in the efficiency of cardiac differentiation were observed between GLA-null CMs and H9 CMs, which suggested that the GLA deficiency did not significantly affect the differentiation of hESCs to CMs. The accumulation of Gb3 in CMs is the most prominent hallmark of FD-associated cardiomyopathy. Compared with H9 CMs, GLA-null CMs accumulated more Gb3, as was demonstrated by transmission electron microscopy (Figure 3A) and thin layer chromatography (Figure 3B). GLA protein was not expressed in #19 and #27 GLA-null CM clones at day 60 of differentiation (Figure 3C). Cardiac hypertrophy is often accompanied by reactivation of fetal genes which are active during fetal cardiac development and quiescent in adult hearts [22]. We measured the expression level of several of such genes by qRT-PCR and showed that atrial natriuretic peptide (ANP), B-type natriuretic peptide (BNP), but not troponin T (TnT) were upregulated in GLA-null CMs compared to H9 CMs. In addition, GLA-null CMs were characterized by shifted balance between α- and β-cardiac myosin heavy chain (MYH6/MYH7 ratio) expression, which is a common response to cardiac injury and a hallmark of cardiac hypertrophy [23] (Figure 3D). Immunofluorescent staining of cardiac troponin T (cTnT) revealed that GLA-null CMs were of significantly larger size (26% ± 9.7%, *p* < 0.001) as compared to the control H9 CMs (Figure 3E,F), which was consistent with the phenotype of FD-associated cardiomyopathy. In summary, we have shown that CRISPR/Cas9-edited hESC-derived GLA-null CMs recapitulated the typical phenotype features of FD-affected CMs and, therefore, could be a useful model to study the FD-associated cardiomyopathy.

### 3.4. Proteomic Analysis of GLA-Null CMs

**Figure 4:**
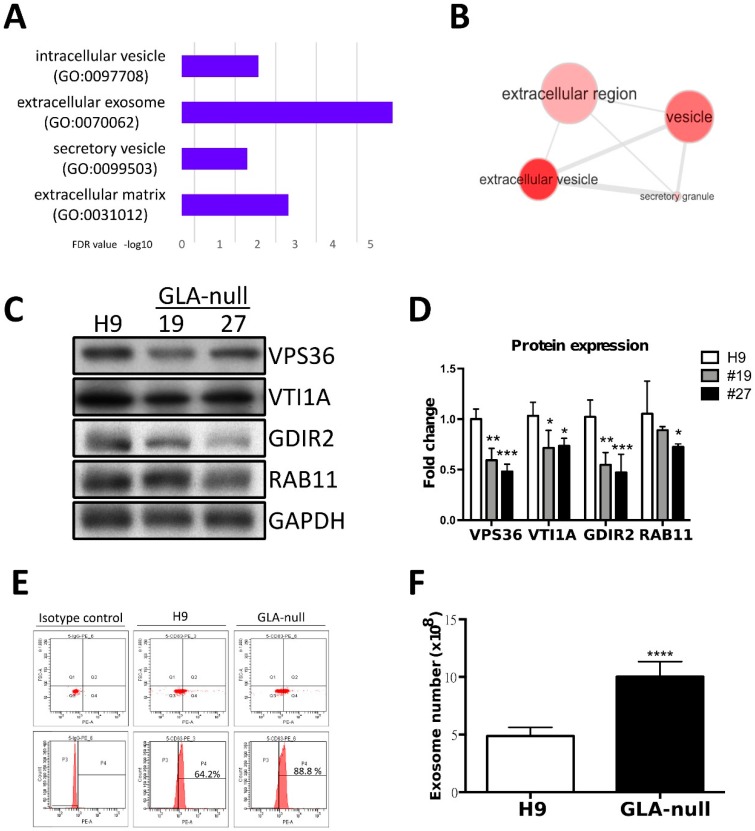
Proteomic analysis of GLA-null CMs. (**A**) The most enriched cellular component GO terms in the list of 60 genes downregulated in GLA-null CMs. (**B**) Visualization of the most enriched cellular component GO terms using REVIGO software. (**C**) Western blot showing expression of the mediators of vesicular trafficking in GLA-null CMs #19 and #27 as compared to wild type control (H9). GAPDH served as a loading control. (**D**) Quantitation of expression of proteins annotated as the mediators of vesicular trafficking. The data are presented as mean ± standard deviation error bars from three independent experiments. (**D**) Flow cytometric analysis of CD63 expression in H9- and GLA-null CM-derived exosome isolated with magnetic beads directly from cell culture medium. (**E**) Quantification of exosome numbers in the supernatant from H9 and GLA-null CMs.

To further investigate the potential mechanism of FD-related cardiomyopathy, we used proteomic analysis. We focused our analysis to the genes that where downregulated by *GLA* knockout, i.e., whose function was negatively affected. By applying LC-MS/MS, we identified 60 proteins downregulated in GLA-null CMs as compared to H9 CMs with a false discovery rate (FDR) below 0.01. By performing gene ontology (GO) analysis using FatiGO software [24], we found that these 60 proteins were significantly enriched in cellular component GO terms related to extracellular vesicle transportation, secretion, and exocytosis (Figure 4A,B). Among the downregulated proteins involved in regulated exosome release were Ras-Related Protein Rab-11 (RAB11), Rho GDP-dissociation inhibitor 2 (GDIR2, ARHGDIB), VPS36 and VTI1A (Supplementary Table S4). The levels of these proteins were validated and quantified by western blotting; they were shown to be downregulated in GLA-null CMs (Figure 4C,D).

### 3.5. GLA-Null CMs Secrete More Exosomes Than H9 CMs

Given the fact that the proteome analysis revealed changes in proteins involved in vesicle secretion, we further investigated whether the exosome biogenesis was affected in GLA-null CMs. The exosomes were isolated from the culture medium, and their identity was confirmed by electron microscopy. Multivesicular bodies were observed and exhibited the typical characteristic cup-shaped morphology (Supplementary Figure S4A) and size (diameter between 50 and 150 nm). Exosome size was measured by the Nanosight tracking system (Supplementary Figure S4B) and their identity was confirmed by detecting expression of exosomal markers TSG101 and CD63 (Supplementary Figure S4C). Quantification of exosome though CD63 PE-conjugated fluorescence by flow cytometry analysis revealed that at higher CD63 positive levels in exosomes derived from GLA-null CMs compared to H9 CMs (Figure 4D). Next, we investigated the number of isolated exosomes from H9 and GLA-null CMs. Interestingly, GLA-null CMs produced significantly larger number of exosomes than H9 CMs (10.8 × 10^8^ vs. 5.1 × 10^8^, *p* < 0.001) (Figure 4E). These results indicate that GLA-null CMs increased the production and secretion of exosomes. 

### 3.6. Vesicle Turnover Impairment Induces Cardiotoxicity in GLA-Null CMs

**Figure 5:**
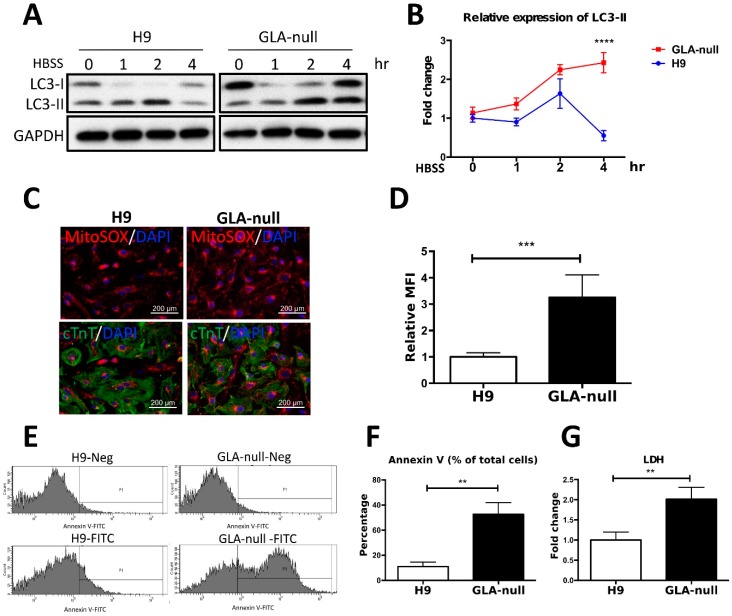
Vesicle turnover impairment induces cardiotoxicity in GLA-null CMs. (**A**) Western blot showing expression of LC3 protein isoforms (LC3-I and LC3-II) in a time course of induction of autophagy with HBSS medium. GAPDH was used as a loading control. (**B**) The expression levels of LC3-II were measured by using ImageJ and the quantification results presented as mean ± standard deviation error bars from three independent experiments. (**C**) Staining of mitochondrial ROS in GLA-null CMs and H9 control CMs with MitoSOX Red. (**D**) MitoSOX Red fluorescence intensity quantified by flow cytometry presented as mean ± standard deviation from three independent experiments. (**E**) Flow cytometry analysis of annexin V-positive cells in populations of GLA-null and H9 CMs. (**F**) Quantitation of annexin V-positive cells. (**G**) The level of LDH secreted by H9 and GLA-null CMs. The data are presented as a fold change relative to H9 control. These data are presented as mean ± standard deviation error bars from three independent experiments.

Several studies have shown that the molecular machinery and regulatory mechanisms are shared between exosome biogenesis and autophagy [25,26], suggesting that these two processes are intimately linked. Since the proteomic analysis revealed the downregulation of components of vesicular trafficking machinery in GLA-null CMs, we hypothesized that dysfunctional autophagy pathway may underlie the impairment of cellular homeostasis in FD-related cardiomyopathy. To investigate whether autophagy flux impairment plays a role in cardiac phenotype of FD, we used western blotting to analyze the expression of autophagy markers LC3-I and LC3-II. The levels of these proteins were monitored in a time course of four hours after induction of autophagy with HBSS medium, and it was revealed that LC3-II accumulated by two hours after autophagy induction in both H9 and GLA-null CMs indicating to initiation of autophagy flux (Figure 5A,B). However, after four hours of autophagy induction, LC3-II levels dropped significantly in H9, but remained high in GLA-null CMs, signifying the digestion of autophagosome by lysosomes in the former and the block of autophagy flux in the latter (Figure 5A,B). Previous studies have indicated that undigested material within the autophagosome, including dysfunctional mitochondria, may be a source of free radicals, which in turn can result in cellular dysfunction and apoptosis [27,28]. Mitochondrial integrity and turnover play an important role in CM bioenergetics and function. MitoSOX Red staining demonstrated significant activation of mitochondrial superoxide production in GLA-null CMs (Figure 5C). The quantification of the MitoSOX Red staining intensity indicated that mitochondrial superoxide production in GLA-null CMs was 3.4-fold higher than that in H9 CMs (Figure 5D). To assess CM apoptosis, annexin V assay was used. GLA-null CMs displayed 4-fold increase in staining with annexin V in comparison with H9 CMs (Figure 5E,F). Furthermore, we observed significant 2-fold increase of lactate dehydrogenase (LDH), a marker of necrosis, in the supernatant derived from GLA-null CMs (Figure 5G). Collectively, our results demonstrate that GLA-null CMs were characterized by autophagy impairment and active mitochondrial ROS production that caused apoptosis and necrosis.

## 4. Discussion

The lysosome storage dysfunction (LSD) is the major factor in etiology of inherited hypertrophic cardiomyopathy, including FD; however, the underlying mechanisms of FD-associated cardiomyopathy are not fully understood. In order to expand the therapeutic strategies for FD, the construction of in vitro disease models using primary human cells is essential and unavoidable. Whereas the patients’ cells can be used to directly model the effects of drugs on humans, their availability and capacity for expansion are limited and finite compared to in vitro derived cell lines, especially for vital organs such as heart and brain. These drawbacks restrict the capacity of these models to faithfully simulate human disease. By comparison, ESCs can sidestep these limitations and thus provide a powerful and versatile tool for disease therapy, as well as basic research. Combined with the advancements in genome editing technology though CRISPR/Cas9, it is now possible to model human diseases in a physiologically, pathologically, and genetically relevant manner.

Mass spectrometry-based proteomics has been recognized as a powerful tool with a potential to uncover detailed changes in protein expression [29]. To date, most of the proteomics studies performed on FD patients examined FD-affected renal tissue or plasma; however, few studies of protein expression have used FD-affected human heart tissue [30,31]. Although it has been revealed that Gb3 accumulation induces endothelial KCa3.1 degradation in *Gla*-knockout mice through clathrin and Rab5C, which are the critical components of endosome maturation machinery [32], the proteomic profiling performed in our study revealed that *GLA* knockout led to the downregulation of Rab GTPases, including RAB11 subfamily, which are involved in recycling from an endosomal compartment to the plasma membrane, and was shown to contribute to exosome secretion in neuronal cells [33], although the molecular mechanism of RAB11 function in exosome secretion has yet to be deciphered, especially regarding its downstream effectors. On the other hand, our proteomic profile also revealed downregulation of Rho GDP-dissociation inhibitor 2 (GDIR2) in GLA-null CMs. This observation is consistent with previously published clinic proteomic profiles, where PBMCs isolated from FD patients were analyzed, and among the downregulated proteins were calnexin, Rho GDP-dissociation inhibitor 1 (GDIR1), Rho GDP-dissociation inhibitor 2 (GDIR2), chloride intracellular channel protein [34]. Rho GDIs play an important role in regulating Rho GTPases, which are members of the Ras superfamily of GTP binding proteins that participate in the regulation of cytoskeleton and other cellular functions including proliferation, differentiation, and apoptosis [35,36]. Rho GDI is ubiquitously expressed and binds to all Rho family proteins, including RhoA [37]. The small G-protein RhoA regulates the actin cytoskeleton, and its involvement in cell proliferation has also been established. In cardiomyocytes grown in vitro, RhoA induces hypertrophic cell growth and gene expression [38,39]. In vivo, however, cardiac-specific overexpression of RhoA leads to development of heart failure [40,41]. These correlated evidences suggest that exosome secretion regulated by Rab GTPase/RhoGDI signaling pathway may utilize as a target for the potential therapeutic strategy for FD-associated cardiomyopathy. 

FD is characterized by failures of cellular autophagy associated with accumulation of glycogen granules and intracytoplasmic vacuoles that contain autophagic material. Impairment of autophagic flux in FD, which was manifested as defects of autophagosome maturation in renal endothelial cells and mesangial cells [42,43]. Autophagy is an evolutionary conserved process of self-degradation of cellular components by autophagosomes, which are delivered to the lysosomal machinery. Several studies have shown that starvation-induced autophagy reduces exosome secretion due to the fusion of multivesicular bodies with autophagic vacuoles [44]. In contrast, cellular stresses, such as senescence and ER stress, increase exosome secretion [45,46]. It is not clear why cells respond to stress by releasing more exosomes, but this could be an alternative way of eliminating waste products. The secreted exosomes may be targeted to and degraded by phagocytes, but they may also have other destinations. Exosomes secreted as waste are likely to affect neighboring cells and possibly induce pathological conditions. Another possibility is that cells may communicate with neighboring cells about intracellular stress by increasing exosome release. Therefore, preventing waste accumulation and rescuing the autophagic ability in FD-affected CMs may be utilized as another therapy approach for FD cardiomyopathy. It has been observed that exosomes derived from CMs harbor a variety of mRNAs, miRNAs and proteins, which may be transferred to the adjacent endothelial cells and modulate their function [47]. Interestingly, exosomes derived from ESCs/iPSCs were shown to possess regenerative power on CMs by augmenting and modulating endogenous repair mechanisms [48,49]. Emerging evidence from the studies of normal development, as well as multiple disease studies, revealed that exosome secretion and autophagy act in a coordinated manner to maintain homeostasis through lysosomal degradation and/or release of cellular cargo [50]. Therefore, considering the role of exosomes in physiological and pathological conditions, strategies that interfere with the release of exosomes and impair exosome-mediated cell-to-cell communication could potentially be exploited therapeutically in FD cardiomyopathy.

The objective of the present study was to explore the influence of Gb3, a lysosomal glycolipid accumulating in FD-affected cells, on modulation of cellular vesicle cycling and the possible mechanism underlying cardiomyopathy. We demonstrated that disruption of *GLA* with CRISPR/Cas9 resulted in the complete ablation of GLA protein expression in hESCs. Results from this study may provide mechanistic insights into how Gb3 accumulation modulates vesicles formation, particularly the autophagy flux in CMs. Such information may be extremely important for potential application in prevention and intervention of adverse effects of FD-associated cardiomyopathy.

## 5. Conclusions

To summarize, in the present study we show that CRISPR/Cas9-mediated *GLA* knockout of hESC-derived CMs can serve as an in vitro FD model for studying hypertrophic cardiomyopathy. Here, we adopted CRISPR/Cas9-mediated genomic editing to successfully generate GLA-deficient hESC clones (Figure 1). These GLA-deficient hESC clones displayed the properties of pluripotency, and were differentiated into CMs, which exhibited the typical biochemical and pathological abnormalities of FD including ablated GLA expression, enlarged cellular size, increased expression of cardiac hypertrophy genes and Gb3 accumulation. Therefore, these GLA-null CMs clones recapitulated the typical characteristics of FD-associated cardiomyopathy (Figure 2 and Figure 3). To determine the global protein expression changes involved in the hypertrophic response to GLA deficiency, we used proteomic analysis and demonstrated markedly reduced expression of proteins involved in cytoskeleton dynamics and secretion of extracellular vesicles (Figure 4). Since exosome biogenesis and autophagy are highly interrelated processes, we have shown that *GLA* knockout led to autophagic impairment, which resulted in increase of mitochondrial ROS production and cell death (Figure 5). Additional investigation of the role of autophagy in FD is highly promising for the development of novel chemical modulation-based therapeutic approaches that may be more economically viable than traditionally-used ERT.

## Supplementary Materials

Supplementary materials are available online at https://www.mdpi.com/2073-4409/8/4/327/s1.

## Abbreviation

LSD	lysosomal storage disease
FD	Fabry disease
Gb3	globotriaosylceramide
α-Gal A	α-galactosidase A
ERT	enzyme replacement therapy
CRISPR	Clustered regularly interspaced short palindromic repeats
sgRNA	single-guide RNA
Cas9	CRISPR-associated protein
KO	knockout
hESCs	human embryonic stem cells
CMs	cardiomyocytes
MVEs	multivesicular endosomes
