# Supplementary material for: Generation of GLA-Knockout Human Embryonic Stem Cell Lines to Model Autophagic Dysfunction and Exosome Secretion in Fabry Disease-Associated Hypertrophic Cardiomyopathy
Source: Cells. 2019 Apr 8;8(4):327. doi: 10.3390/cells8040327 (PMC6523555; doi:10.3390/cells8040327)
Supplement: Supplementary file 1 [file cells-08-00327-s001.pdf]

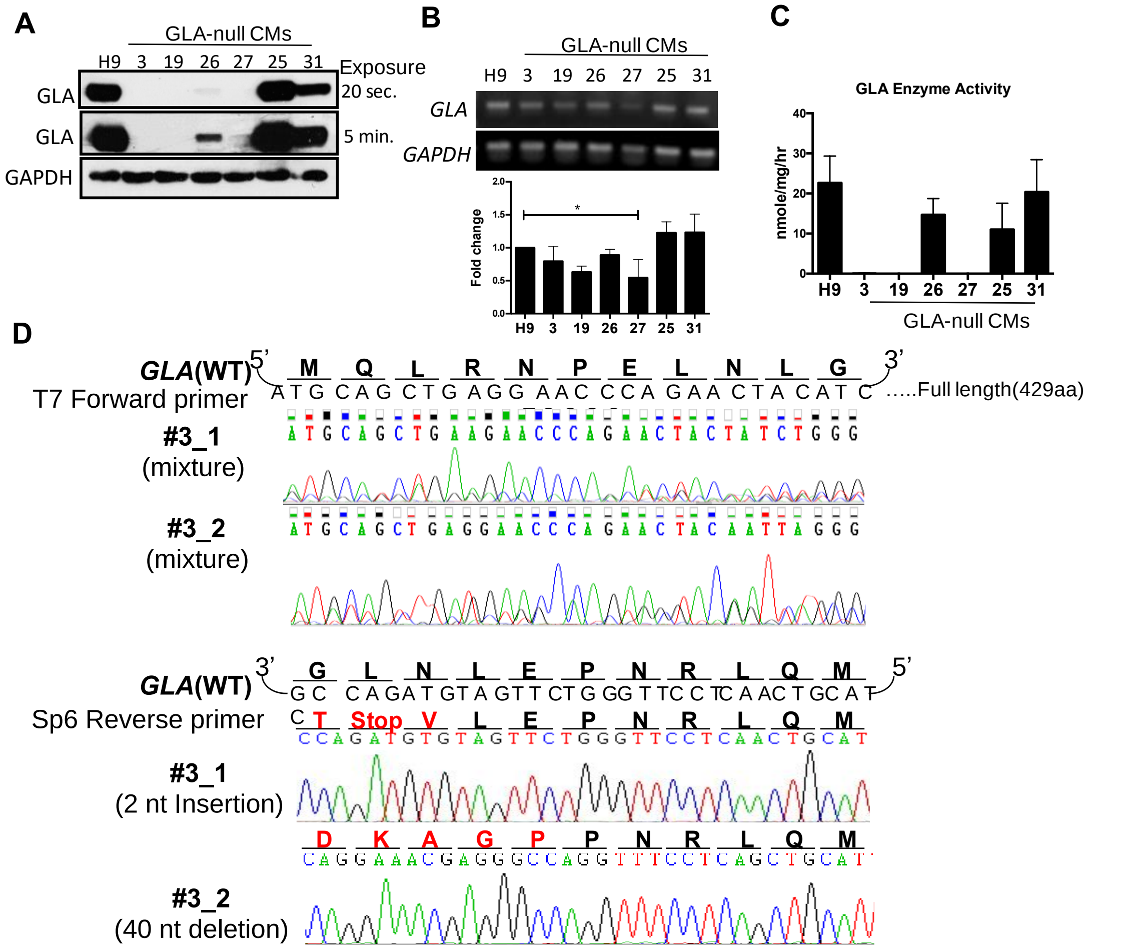

Supplementary Figure 1. (A) GLA protein expression level in GLA-null clones #3, 19, 26, 27, 25, and 31 derived CMs Exposure in short time:20 sec and longer time:5 mins. (B) mRNA level in GLA-null clones #3, 19, 26, 27, 25, and 31 derived CMs. (C) GLA enzyme activity in GLA-null clones #3, 19, 26, 27, 25, and 31 derived CMs. (D) Sanger sequencing analysis revealed heterogeneous population in clone #3.

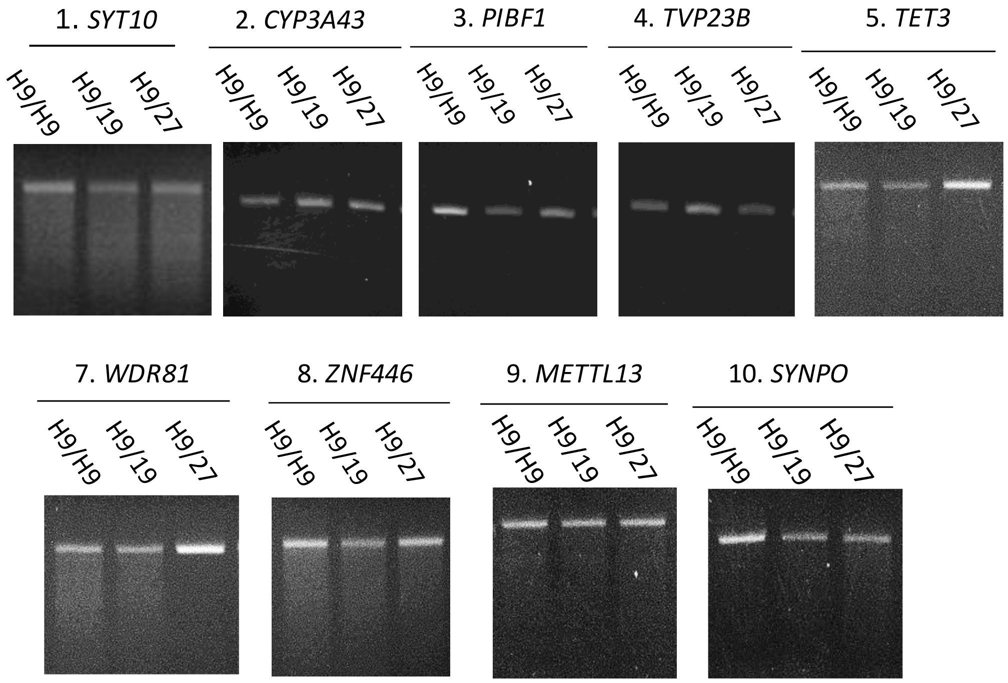

Supplementary Figure 2. T7E1 digestion assay validating the absence of CRSIPR/Cas9-induced mutations in the predicted off-target genes. T7E1 cleavage assay showing the absence of mismatch cleavage products.

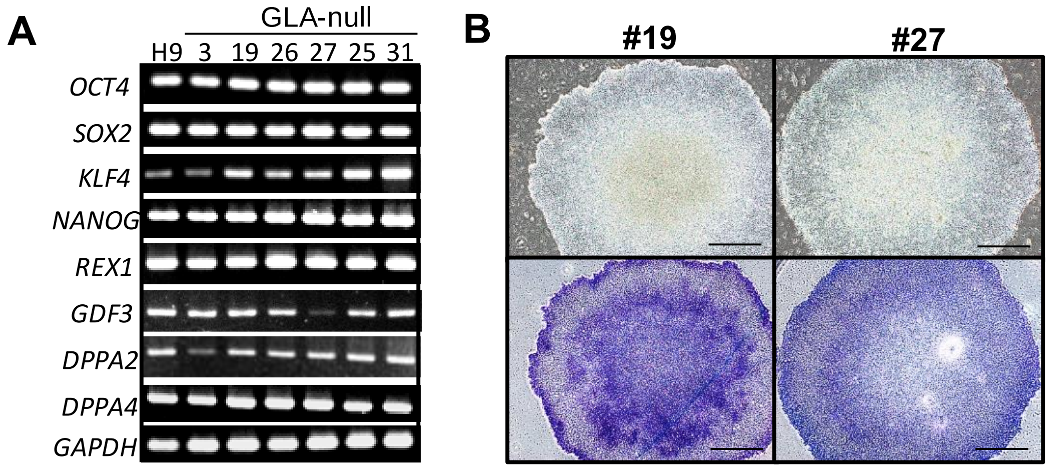

Supplementary Figure 3. Characterization of CRISPR/Cas9-edited GLA-null hESC clones. (A) RT-PCR analysis of expression of pluripotency-associated genes in CRISPR/Cas9-transfected hESC clones, including GLA-null clones #19 and #27. Untransfected parental hESCs (H9) served as a positive control. (B) Morphology and alkaline phosphatase activity of GLA-null hESC clones #19 and #27.

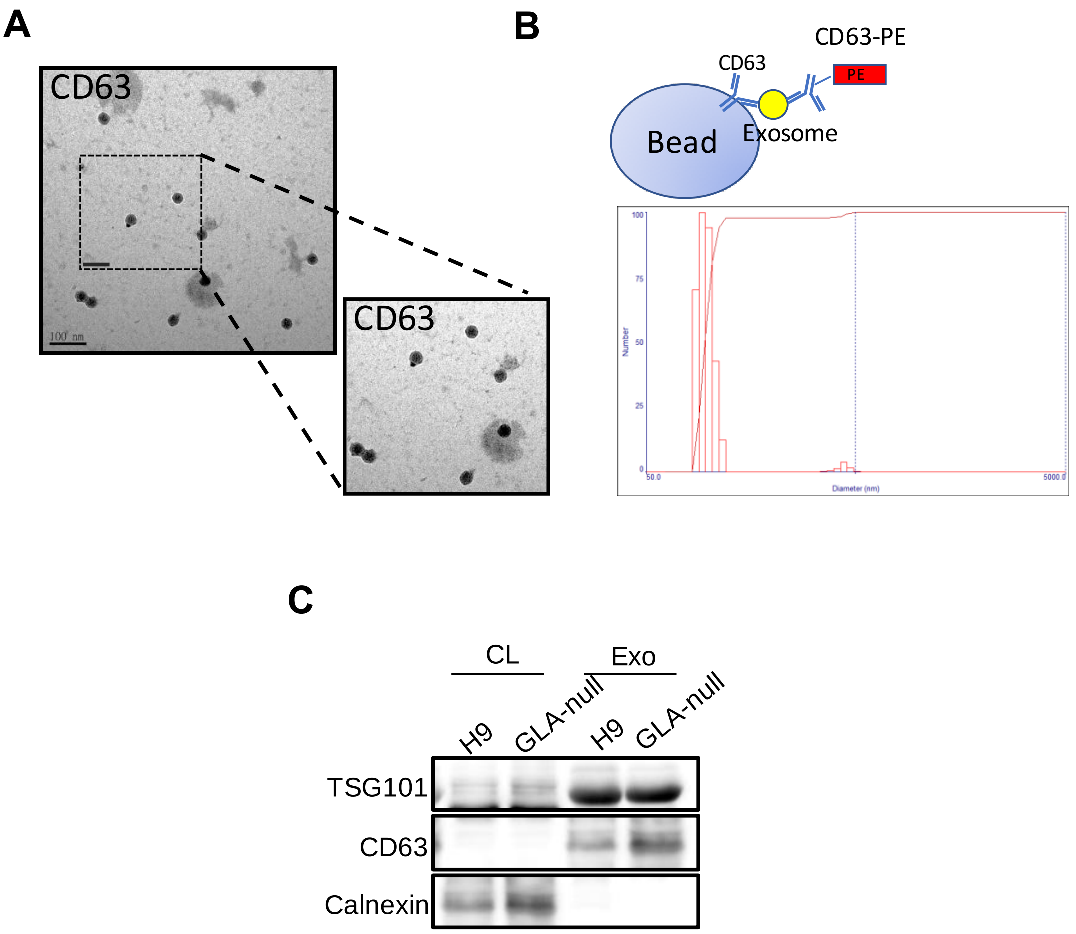

Supplementary Figure 4. Characterization of CM-derived exosomes. (A) Representative electron microscopy images of isolated exosomes. Scale bar: 100 uM. (B) CD63 PE-conjugated dynabeads isolated and identified distribution of CMs derived exosome sizes though Nanosight tracking system with a diameter range of 50–100 nm. (C) Western blot showing expression of exosomal markers TSG101 and CD63 in cell lysate (CL) and exosome-containing culture medium (Exo) of H9 and GLA-null CMs. Calnexin used as CL positive control.

Supplementary Table 1. List of primers used to amplify the predicted off-target gene loci.

	Gene No.	Gene Name	Chromosome Site	Primer Sequence	Product Size
1	NM_198992	*SYT10*	chr12:-33559846	TAGCATGGGCACAGAACCTG<br>TTCAGCATTAGGTGCCTGAATTA	684
2	NR_103869	*CYP3A43*	chr7:+99454520	CCAGGAAGTTGTGTCCAAAGG<br>CAGTGTGTCTCCTGATTGGATG	699
3	NM_006346	*PIBF1*	chr13:+73467918	AGCAAGCTGGGAAACAATGC<br>CCCTCCCAGAAACATGGTGT	867
4	NM_016078	*TVP23B*	chr17:+18702311	TCCTTGATCATTGGTAGGAAAGGT<br>TTGTTAGGCAGCAATAGGTTACCA	739
5	NM_144993	*TET3*	chr2:-74328546	GTCCCCCAAGAGGACTAACG<br>GGGCACACTCGATGAGGAT	674
7	NM_152348	*WDR81*	chr17:-1639089	GCGGTGAGTTGGGGGATTAG<br>AGCACCATGAAGCCTGAGGA	660
8	NM_017908	*ZNF446*	chr19:-58992296	ACCGCAAGAGCCACACAG<br>AACTGCCTATTTCCCGACCA	679
9	NM_014955	*METTL13*	chr1:+171759733	GCACACTGCTGCCAGTAACC<br>CAGGGACTTCAGGTGAAAACG	736
10	NM_001166208	*SYNPO*	chr5:-150031531	CCTGGATTCTAACAGACCAACTGC<br>GAGCAGGCCCACTCCACTT	699

Supplementary Table 2. Sequences of the primers used to analyze stemness markers byRT-PCR

Name	Sequence	Predicted size
OCT4	F_CTTCAGGCACTGTGTTCATTG<br>R_TTTGGCTGAACACCTTCCCA	672 bp
SOX2	F_GCCCTGCAGTACAACTCCAT<br>R_TTCCTGCAAAGCTCCTACCG	735 bp
KLF4	F_AGTTTCCCGACCAGAGAGA<br>R_ACGCGAACGTGGAGAAAGAT	667 bp
NANOG	F_GAAGACAAGGTCCCGGTCAA<br>R_GGATTCAGCCAGTGTCCAGA	709 bp
REX1	F_GTGGGCCTTATGTGATGGCT<br>R_TGCGTTAGGATGTGGGCTTT	759 bp
GDF3	F_ GTTTGTGTTGCGGTCAGTCC<br>R_ CTTGGGGGCAATGATCCACT	361 bp
DPPA2	F_CCGTCCCCGCAATCTCCTTCCATC<br>R_ATGATGCCAACATGGCTCCCGGTG	606 bp
DPPA4	F_TAGCACAGCAAAAGAGGCCA<br>R_TGCATGGCCCATAAACAGGT	635 bp
GAPDH	F_AGAAGGCTGGGGCTCATTTG<br>R_AGGGGCCATCCACAGTCTTC	258 bp

Supplementary Table 3. Antibodies used in this study

Target	Source	Catalog number
GLA	GeneTex	GTX101178
NANOG	Cell Signaling Technology	#4903

OCT4	Cell Signaling Technology	#2750
TRA-1-81	Abcam	Ab16289
TRA-1-60	Abcam	Ab16288
Nestin	Cell Signaling Technology	#4760
alpha-SMA	Cell Signaling Technology	#19245
AFP	Cell Signaling Technology	#4448

Supplementary Table 4. List of the differentially downregulated proteins involved in regulated exosome release.

Uniprot	Protein Name	Gene Name
Q13636	Ras-related protein Rab-11	RAB11
Q86VN1	Vacuolar protein-sorting-associated protein 36	VPS36
Q13017	Rho GTPase-activating protein 5	ARHGAP5
P52566	Rho GDP-dissociation inhibitor 2	ARHGDIB
Q9NP61	ADP-ribosylation factor GTPase-activating protein 3	ARFGAP3
P35475	Alpha-L-iduronidase	IDUA
Q96AJ9	Vesicle transport through interaction with t-SNAREs homolog 1A	VTI1A
Q14894	Ketimine reductase mu-crystallin	CRYM
P07996	Thrombospondin-1	THBS1
Q02952	A-kinase anchor protein 12	AKAP12
Q9UJA5	tRNA-methyltransferase non-catalytic subunit TRM6	TRMT6
Q96EY4	Translation machinery-associated protein 16	TMA16
Q86SZ2	Trafficking protein particle complex subunit 6B	TRAPPC6B

Supplemental Reference

1. Labuhn, M., et al., Refined sgRNA efficacy prediction improves large- and small-scale CRISPR-Cas9 applications. Nucleic Acids Res, 2018. 46(3): p. 1375-1385.
